# Lipid Production from *Nannochloropsis*

**DOI:** 10.3390/md14040061

**Published:** 2016-03-25

**Authors:** Xiao-Nian Ma, Tian-Peng Chen, Bo Yang, Jin Liu, Feng Chen

**Affiliations:** Institute for Food and Bioresource Engineering, College of Engineering, Peking University, Beijing 100871, China; maxiaonian126@126.com (X.-N.M.); tianpeng_chen@163.com (T.-P.C.); ly_mikeyang@163.com (B.Y.)

**Keywords:** lipid accumulation, *Nannochloropsis*, cultivation, triacylglycerol, eicosapentaenoic acid, genetic engineering

## Abstract

Microalgae are sunlight-driven green cell factories for the production of potential bioactive products and biofuels. *Nannochloropsis* represents a genus of marine microalgae with high photosynthetic efficiency and can convert carbon dioxide to storage lipids mainly in the form of triacylglycerols and to the ω-3 long-chain polyunsaturated fatty acid eicosapentaenoic acid (EPA). Recently, *Nannochloropsis* has received ever-increasing interests of both research and public communities. This review aims to provide an overview of biology and biotechnological potential of *Nannochloropsis*, with the emphasis on lipid production. The path forward for the further exploration of *Nannochloropsis* for lipid production with respect to both challenges and opportunities is also discussed.

## 1. Introduction

Fossil fuels, the primary global energy source, are widely recognized as unsustainable, and renewable forms of energy including biofuels are highly sought after [1]. It is essential to improve the production of biodiesel, which is a promising renewable and safe alternative to petroleum oils. Owing to the low content of sulfur and high ratio of oxygen, biodiesel has reduced CO and SO_2_ discharge as compared to petroleum [2]. Currently, plant oils and animal fats serve as the main sources for biodiesel production, but biodiesel produced from these feedstocks cannot realistically replace the petroleum-derived transport fuels in the foreseeable future [1]. By contrast, microalgae are emerging as the next-generation biodiesel feedstock and have the potential to meet the existing demand for transportation fuels [1,3]. Microalgae are photosynthetic microorganisms responsible for at least 32% of global photosynthesis and nearly half of the atmospheric oxygen [4,5]. Advantages of using microalgae for biodiesel production include but are not restricted to: (1) Microalgae have high photosynthetic efficiency; (2) They do not compete with crops for arable land; (3) CO_2_, nitrogen and phosphate are utilized while culturing microalgae, leading to mitigation of greenhouse effect and water pollution. Approximately 1.8 kilograms CO_2_ are required to produce one kilogram biomass [6]; (4) Microalgae grow fast and can accumulate up to 80% lipid content of biomass [7]; Microalgal storage lipids are mainly triacylglycerols (TAGs), which can be used as the feedstock for biodiesel conversion through transesterification reaction with methanol; (5) Biodiesel from microalgal oil is comparable to the standard biodiesel in terms of the key properties [8].

Fatty acids are classified into saturated, monounsaturated and polyunsaturated fatty acids (PUFAs). PUFAs are important bioactive substances, consisting of two principle families: ω-3 and ω-6 series, with their first double bond at carbon atom number 3 and 6, respectively [9]. There is evidence that intake of ω-3 PUFA could benefit human health and promote animal growth [10,11]. Eicosapentaenoic acid (EPA, C_20_H_30_O_2_) and docosahexaenoic acid (DHA, C_22_H_32_O_2_) are the most important ω-3 PUFAs showing significant positive effects on heart disease prevention, anti-inflammatory activity, brain development and vision health [12]. It has been recommended that daily intake of 250 mg (primary prevention) to 2 g (secondary prevention) of EPA and DHA can prevent coronary heart disease and other aging related diseases [11].

At present, seafood is generally used for commercial production of both DHA and EPA. However, microalgae are the primary producers of ω-3 PUFAs and fish usually obtain EPA via bioaccumulation from the food chain [12]. The global market of fish oils is limited because of the stagnant production of world fishery, chemical contamination from the severely polluted ocean, unpleasant odor, unacceptability for vegetarians, *etc.* [13,14]. Marine microalgae are the primary producers of ω-3 PUFAs and have been considered as promising alternative to fish oils for PUFA production.

The microalgal genus *Nannochloropsis* has been receiving ever-increasing research interest owing to its ability to synthesize not only neutral lipids for biodiesel production but also EPA for functional food [15,16]. In the present review, we aim to summarize the current status and perspectives of lipid production from *Nannochloropsis*, in terms of culture techniques and mechanism regulating oil synthesis at the cellular level.

## 2. Taxonomy and Morphology of *Nannochloropsis*

**Figure 1:**
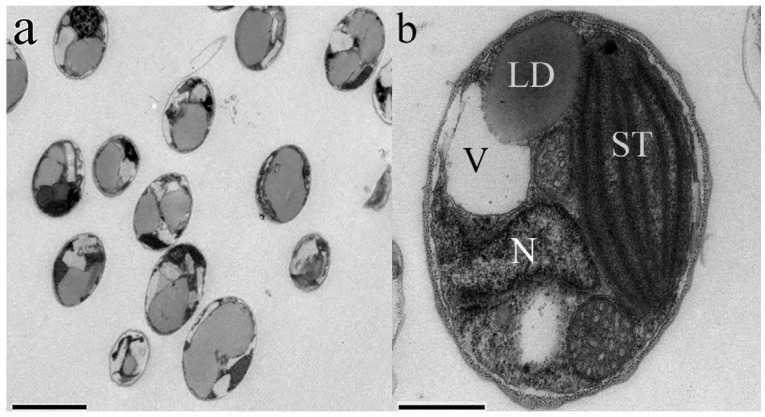
Micrographs of typical *N. oculata* representing (**a**) the morphology; and (**b**) different organelles. LD, lipid droplet; N, nucleus; ST, stacks of thylakoids; V, vacuoles. Bars = 2 μm for (**a**) and 0.5 μm for (**b**).

**Figure 2:**
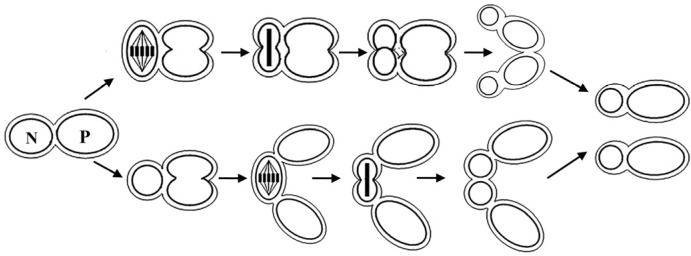
Schematic diagrams of the replication cycle of *N. oculata*. The order of the nuclear (N) and plastid (P) division is not strictly fixed. Nuclear division precedes the plastid division (**upper**); or vice versa (**lower**) [18].

*Nannochloropsis* is a genus of unicellular and nonmobile marine microalgae belonging to: Phylum Heterokontophyta, Class Eustigmatophyceae and family Eustigmataceae. It was first termed by Hibberd [17], comprising six known species: *Nannochloropsis gaditana*, *Nannochloropsis granulata*, *Nannochloropsis limnetica*, *Nannochloropsis oceanica*, *Nannochloropsis oculata* and *Nannochloropsis salina*. The cell is simple in morphology with diameters varying from 2 to 8 μm, and it has plastids similar to plant cells (Figure 1). The chloroplast with clearly visible stacks of thylakoids is in close proximity to the nucleus. The lipid droplet (LD) functions as an energy depot, which can be augmented in size under stressful conditions (Figure 1b). The cell division of *N*. *oculata* was first reported by Murakami and Hashimoto [18], which revealed the existence of a continuum between nucleus and plastid throughout the cell cycle (Figure 2).

## 3. *Nannochloropsis* as a Promising Cell Factory for Lipid Production

**Figure 3:**
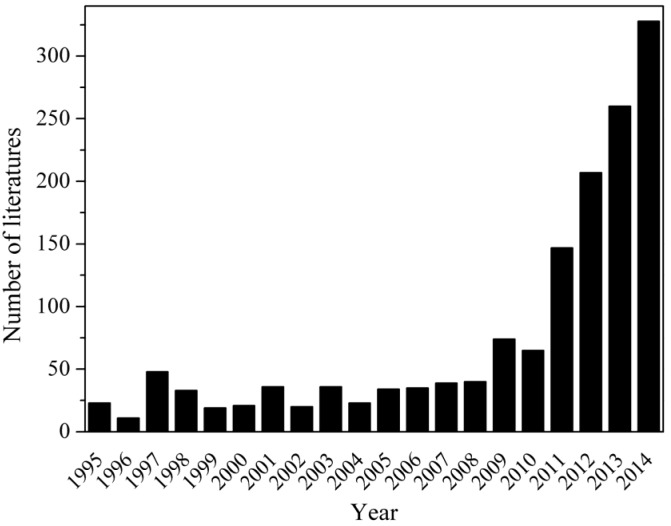
Publications on *Nannochloropsis* in the past 20 years (1995–2014) (from Web of knowledge).

It is general accepted that high growth rate, considerable lipid content, proper fatty acid composition, good environmental adaptability and contamination resistance are preferred traits when screening microalgal candidates for lipid production. *Nannochloropsis* has been receiving ever-increasing research attention, which is witnessed by a boom in the number of journal publications in the past years (Figure 3).

### 3.1. Cell Growth and Lipid Accumulation

*Nannochloropsis* is considered as a potential oleaginous model microalga because of the great photosynthetic efficiency, high lipid productivity, well established genetic toolbox and relatively mature technology for outdoor cultivation systems on a large scale [6,19,20]. In general, the specific growth rate of reported *Nannochloropsis* strains ranged from 0.11 to 0.21 per day, except that *N. limnetica* CCMP505 showed a relatively low growth rate (0.07 per day), according to the investigation on growth kinetics [16]. In the investigated *Nannochloropsis* species, only *N*. *limnetica* was cultivated in fresh water, which may result in its strange phenomenon in cell growth [16]. Lipids are important structural and functional parts of microalgae, including polar membrane lipids and neutral lipids. Under optimal growth conditions, microalgae synthesize fatty acids principally for esterification into glycerol-based membrane lipids, which constitute about 5%–20% of the cell dry weight (DW); under unfavorable or stressed conditions, microalgal lipid biosynthesis is channelled into neutral lipids mainly in the form of TAGs (20%–50% DW) [3]. As indicated by Table 1, *Nannochloropsis* has a lipid content ranging from 37% to 60% (of DW), higher than other microalgal strains, indicative of the superiority of *Nannochloropsis*. Doan *et al.* [21] conducted a comprehensive high-throughput screening study and the results suggested that three *Nannochloropsis* strains out of 96 tested strains were the best feedstocks for biodiesel. *N. oceanica* IMET1 was considered as another excellent strain for lipid production, with a lipid productivity of 158 mg/L·day and TAG production of 1.67 g/L [16].

### 3.2. Fatty Acid Profile

Ideal microalgal candidates for biodiesel production should have suitable fatty acid composition in addition to high lipid content. Fatty acid profile affects the properties of biodiesel, such as heat of combustion, lubricity, viscosity, low-temperature properties and oxidative stability [8]. Most of the vegetable oils have fatty acid profiles in the typical C16–18 range and large proportions of saturated fatty acids, leading to a biodiesel fuel that likely possesses a cloud point >0 °C [8]. As shown in Table 2, the fatty acid distribution varies across *Nannochloropsis* strains, with C16, C18 and C20 being the majority. Of the 6 known species, *N. oculata*, *N. gaditana* and *N. salina* contain mainly C16 fatty acids while *N. limnetica* contains mainly C18 fatty acids (Table 2). The great difference in fatty acid profile of *N. limnetica* as compared to other strains may be due to the different culture conditions as *N. limnetca* was grown in fresh water instead of sea water [16]. In general, saturated fatty esters are high in cetane number and thus improve the oxidative stability of the biodiesel, whereas unsaturated fatty esters have better low-temperature properties. It has been suggested that monounsaturated fatty ester may act as a balance between oxidative stability and low-temperature properties, and its proportion is considered as an important index to assess the biodiesel quality of microalgal oils [8,16]. In this regard, *N. oculata* and *N. granulata*, which have a higher percentage of monosaturated fatty acids (over 50%, Table 2), are superior to other strains for biodiesel production. As suggested by a recent study [16], the biodiesel properties of *Nannochloropsis* oils including kinematic viscosity, specific gravity, cloud point, cetane number, iodine value, and higher heating value meet the specification established by both US (ASTM D6751) and Europe (EN 14214) standards.

The fatty acid profile of *Nannochloropsis* can be influenced by a variety of factors, including light intensity, light regime, temperature, pH and other environmental stresses. Increasing light intensity led to a decrease in polyunsaturated fatty acids, accompanied by an increase in C16:0 and C16:1 in *Nannochloropsis* sp. [32]. The percentage of saturated fatty acids was higher in cells grown under blue (47%) and red light (53%) than cells grown under white light (37%) in *N. gaditana*, which mainly resulted from the increase in C16:0 and the decrease in C20:5 [33]. Lower temperature favored the unsaturation level of fatty acids, while higher temperature tended to increase the saturation level in *N. salina*, suggesting that the physiological adaptations of microalgae occurred to change membrane fluidity [34].

### 3.3. EPA Production

Microalgae have emerged as promising natural resources for highly unsaturated fatty acids [35]. *Nannochloropsis* is considered as one of the most promising microalgae due to its high yields in EPA [15]. There have been many studies reporting the use of *Nannochloropsis* for EPA production in the past years. As summarized in Table 3, the EPA content varies considerably from 1.1% to 12% of DW across *Nannochloropsis* strains. The highest EPA content (12% DW) was achieved in a *Nannochloropsis* isolate reported by Sharma and Schenk [36], indicating that this strain is a good candidate for EPA production. Nevertheless, it is worth noting that these studies were carried out by individual groups under different culture conditions, making the direct comparison in EPA content difficult. A comprehensive and comparative study remains to be conducted to screen a high performance *Nannochloropsis* strain along with an optimal operational protocol for EPA production.

Various biochemical approaches have been applied to promote the EPA biosynthesis in *Nannochloropsis*. *N. gaditana* can be grown outdoors at dilution rates as high as 0.4 per day under different environmental conditions throughout the year, with this species being tolerant to low temperature (17 °C) and irradiance of more than 500 μmol/m^2^·s in a greenhouse to maximize EPA productivity [37]. Compared to other binary LEDs, the combination of blue and red illumination led to the highest EPA productivity of 14.4 mg/L·day in *N. oceanica* [38]. Differing from the nitrogen limitation conditions favorable for TAG accumulation, a high EPA productivity (7.6 mg/L·day) was obtained by nitrogen-replete cultivation in *N. oceanica*. It could be explained by the fact that EPA was mainly located in glycolipids and phospholipids which accumulated under nitrogen replete conditions, while neutral lipids were the dominant carbon sink under nitrogen-depleted conditions [39].

### 3.4. Waste Water Bioremediation and CO_2_ Biomitigation

The mass consumption of fossil fuels releases a large amount of CO_2_ into atmosphere, leading to many environmental issues including global warming that threaten our ecosystem [1]. The potential environmental benefits of microalgae, such as CO_2_ mitigation and bioremediation of waste water by removing large amounts of nutrients and heavy metals, have attracted much attention recently. The cultivation of *Nannochloropsis* in waste water for nitrogen utilization as well as flue gas for CO_2_ supply has also been investigated in several studies (Table 4). These environmentally beneficial applications may be coupled with lipid production, which represents a possible direction toward cost-effective production of lipids by *Nannochloropsis*.

*N. salina* was reported to be mainly utilized for effluent treatment [43,44,45]. Anaerobic digester (AD) effluent is enriched in both phosphate and ammonium, which is too high to be tolerated by microalgae. Dilution is thus necessary since the inhibitory effects of AD effluent on microalgal growth occurrs. Moreover, other nutrients can be added to achieve a desired recipe required for cell growth. As shown in Table 4, the AD effulent loading of 3% gave rise to the highest lipid content (35% DW), while higher loading up to 18% led to a decline in lipid content [43]. The cell growth of *N. salina* subjected to AD effluent was generally comparable to that of commercial nutrients at the same total nitrogen level [45].

Flue gas serves as an inexpensive carbon source for cultivation of microalgae, and the greenhouse effect can be alleviated at the same time. However, the sulfur and nitrogen oxides in flue gas could reduce the medium pH and thus inhibit the cell growth, indicative of a much more tolerable traits for the candidate microalgae species [46]. Although ambient air was often used as a source of CO_2_ in algal growth studies, the biomass productivity could be increased by bubbling additional CO_2_. Flue gas from the combined heat and power unit of an anaerobic digestion project may be used as an economic CO_2_ source for *Nannochloropsis* growth on a commercial scale [43]. This possibility has been demonstrated by the combined use of municipal wastewater and flue gas in *Nannochloropsis*, which was used to replace nutrients and provide CO_2_, respectively [47].

## 4. Cultivation of *Nannochloropsis*

Generally, microalgae may be cultivated photoautotrophically, heterotrophically and mixotrophically in open ponds or closed bioreactors. To minimize the cultivation expense, it is preferable to rely on freely available sunlight for lipid production despite daily and seasonal variations in light levels [1]. It has been reported that several species of *Nannochloropsis* are successfully cultivated at large scale using natural sunlight by companies such as Solix Biofuels, Aurora Algae, Seambiotic and Proviron [19]. However, the poor light penetration in high cell density may reduce the cell growth, which hinders the application of microalgae for large-scale autotrophic production. *Nannochloropsis* has been explored for high cell density cultivation to improve their possibilities as the biodiesel or PUFAs feedstock in recent years.

### 4.1. Photoautotrophic Growth

Photoautotrophic cultivation is the most common way for growing microalgae due to its low costs and environmental-friendly properties. Open raceway ponds are highly recognized since they are convenient to operate, and cheap to construct and maintain. Energy can be obtained from sunlight, and carbon dioxide is from power plants, facilitating the mitigation of environmental problems [2]. *Nannochloropsis* strains have been cultivated successfully using flue gas in 8000 L indoor open raceway ponds [46]. One local saline tolerant *Nannochloropsis* strain was grown in a 25,000 L raceway pond for fish feed in the Qatari desert [49]. A computational model has been provided to predict the *N*. *salina* growth in systems across varying scales and identify the factors that may affect the algal productivities, which could be helpful for the future optimization of pond designs [50]. The depth of the pond is limited to no more than 30 cm in order to ensure the light penetration. As a consequence, the biomass is relatively limited and generally no more than 1 g/L dry weight was achieved [51]. In addition, the water evaporation and contamination are severe issues associated with the cultivation in open ponds.

Closed bioreactors are more controllable than open ponds and benefit microalgal growth and lipid production. A tubular bioreactor is the most widely used commercial system, with generally 20 cm or less in diameter [1]. They are commonly made of transparent materials, likely polypropylene acrylic or polyvinylchloride pipes with the thickness of a few milimeters, allowing appropriate light absorption [4,52]. However, improvements are to be expected in material lifetime because polyethlene has a relatively short lifetime of about one year [53]. The mixing and agitation of the culture is realized using air-pump forming bubbles, and the illumination can be obtained from sunlight or artificial light [52]. The feasibility of helical-tubular bioreactors by *Nannochloropsis* sp. has been recently demonstrated, reaching a productivity of 1.10–3.03 g/L·day [52]. The used tubular photobioreactor showed advantages in the large ratio of culture volume to surface area, the optimized light penetration depth and the novel automated flow-through sensor providing continuous cell concentration monitoring [52]. A vertical flat-plate photobioreactor was designed, and the effects of reactor dimensions, irradiation and cell concentration on the biomass productivity were evaluated [54]. A short light path is preferred to increase the volumetric productivity and biomass concentration. The distance between the flat-plate reactors should be enlarged properly to avoid mutual shading of the plates [55]. Even though closed photobioreactors show obvious advantages of good controllability, the high costs in construction and sterilization of complex system will hinder their application.

### 4.2. Heterotrophic Growth

Some microalgae can grow heterotrophically with organic carbon source in the absence of light, thus avoiding light limitation or photoinhibition. Both cell growth and biosynthesis of products are significantly affected by nutrients and environmental factors. Carbon sources, typically such as glucose, glycerol and acetate, are mostly indispensable for heterotrophic culture [2]. Vazhappilly and Chen [56] tried to grow *N. oculata* with glucose or acetate, and the results suggested that this strain was unable to utilize these two organic carbon sources for heterotrophic growth. There are only a limited number of reports about heterotrophic growth of *Nannochloropsis* [30,57]. The biomass of *Nannochloropsis* sp. reached to 326 mg/L in heterotrophic culture with glucose, less than that cultured in photoautotrophic culture (392 mg/L) [30]. Similarly, the lipid yield of *Nannochloropsis* sp. was slightly lower in heterotrophic culture compared with that in photoautotrophic culture [57].

### 4.3. Mixotrophic Growth

A major problem hindering the application of microalgae for lipid production is light intensity reduction in high density culture, which can be partially solved by mixotrophic operation [58]. *Nannochloropsis* is capable of growing mixotrophically in the presence of organic carbon sources under illumination conditions. Hence, the cell growth may not strictly rely on light or organic carbon. It was reported that the growth rate and cell density of *N. oculata* could be promoted by adding glucose of 0.1 g/L [58]. Although increasing initial glucose concentration (from 0 to 20 g/L) enhanced the growth of *Nannochloropsis* sp., it reduced the total lipid content [57]. Nevertheless, the lipid yield of *Nannochloropsis* sp. increased from 109.8 to 798.1 mg/L when the initial glucose concentration was increased from 0 to 15 g/L, while a slight decrease of lipid yield was observed at initial glucose concentration of 20 g/L [57].

## 5. Biochemical Engineering Methods Enhancing Lipid Accumulation in *Nannochloropsis*

Microalgae show the capacity of acclimating to different culturing conditions, with changes occurring in morphology as well as physiology. Under optimal growth conditions, algae synthesize fatty acids principally for esterification into glycerol-based membrane lipids, where PUFAs are mainly located. Under unfavorable or stressed conditions, neutral lipids (mainly TAGs) accumulate [3]. The lipid profiles of *Nannochloropsis* are subject to many nutritional and environmental factors including irradiance, nitrogen concentration, salinity, *etc.* It is suggested that the manipulation of culture conditions has the potential to enhance lipid accumulation in *Nannochloropsis*. According to some reported studies, moderate illumination (normally less than 100 μmol/m^2^·s), adequate nutrient components (such as nitrogen and phosphate) and lower salinities (such as 25‰) favored cell growth and the synthesis of EPA. On the contrary, excess light, nutrients starvation and higher salinities (such as 35‰) led to the extensive accumulation of TAGs [29,32,58,59,60,61,62]. In fact, reflections vary differently across species when exposed to the same culturing condition. With respect to *Nannochloropsis*, it is difficult to provide the exact culturing parameters to achieve the best state towards a high TAG/EPA productivity. More efforts are still needed to elucidate the key factors that may influence cellular activities and clarify mechanisms in order to enhance lipid production using biochemical engineering methods.

### 5.1. Irradiance

Irradiance, as a major factor affecting lipid productivity, is closely related to photosynthetic efficiency and carbon assimilation [59,63]. The damage to the photosynthetic antenna system and the photosynthetic II reaction center (PSII) occurs upon exposure to excess light, accompanied by the photobleaching of pigments [60]. While chlorophylls are necessary for light harvesting, and carotenoids are essential for defending oxidative stress [59], both cellular chlorophyll a and carotenoid content decreased as the irradiance level increased [32]. A strong linear relationship was found between total carotenoid content and chlorophyll a percentage, indicating that the changes of pigment content occurred in a highly synchronous manner [61].

*Nannochloropsis* grown under saturating light has a higher biosynthesis of total lipids and carbohydrates compared with that under light-limited conditions [32]. The changes in polar lipid content and composition may result from the decomposed or resynthesized thylakoid membrane lipid in chloroplast where EPA is mainly located [32]. There was a reduction in the percentage of both monogalactosyl diacylglycerol (MGDG) and digalactosyl diacylglycerol (DGDG) when the light intensity was increased from 35 to 550 μmol/m^2^·s [32]. Similarly, a quantitative decrease in phospholipid and glycolipid levels was also observed in freshwater and marine cyanobacteria under high light [60]. The effects of light sources on lipid accumulation have been investigated, showing that the combined blue-red LED led to a higher EPA productivity than other binary LEDs [38]. As expected, when grown under high light conditions that favored TAG production, an increase in the percentage of both C16:0 and C18:1 with a concomitant decline in EPA percentage was found, indicating the obvious relationship between the decomposition of the EPA-enriched thylakoid membrane lipids and enhanced biosynthesis of neutral lipids [61].

### 5.2. Nitrogen

Nitrogen starvation is generally considered as the most effective factor to trigger TAG accumulation [62]. It was reported that a 75% reduction of the nitrogen concentration in the medium led to about 100% increase in the total lipid content in *N. oculata* [64]. In response to nitrogen starvation, the genes coding for light harvesting complex were downregulated and 1213 genes including key carbon fixation, tricarboxylic acid (TCA) cycle, glycerolipid metabolism as well as nitrogen assimilation was upregulated in diatom [65]. During chronic nitrogen starvation, *N. oceanica* evolved a series of physiological strategies to survive long-term nitrogen stress, including pigment alteration, lipid accumulation, reduction in photosynthesis, carbon fixation, and protein synthesis [61].

While neutral lipids are the dominant storage compounds in *Nannochloropsis* under nitrogen-depleted condition, nutrient-replete conditions are beneficial to the biosynthesis of polar lipids. It has been shown that the highest EPA productivity (7.66 mg/L·day) was achieved under nitrogen-replete cultivation, since EPA is mainly present in glycolipids and phospholipids [39].

### 5.3. Salinity

The effects of salinity on cellular physiology cannot be ignored due to the halophilism of some *Nannochloropsis* species. The appropriate salinity range of *N*. *oculata* has been documented between 10‰ and 35‰, and the optimum for growth was 25‰ under nutrient-replete conditions, whereas it grew better at 35‰ following nitrogen starvation [29]. Large-scale cultivation of photoautotrophic microalgae in open ponds is limited mainly due to the contamination of plankton. Nevertheless, this can be reduced by high-salinity cultivation [66]. The biomass was the greatest at the salinities of 22‰ and 34‰, while the highest lipid content was obtained at a salinity of 34‰ in *N. salina*, while the density of invading organisms was lowest at 22‰ [67]. The effect of salinity on lipid accumulation in *N. oculata* was studied and the results suggested that low salinity (25‰) promoted EPA synthesis while the salinity of 35‰ led to the highest lipid productivity (65 mg/L·day) after 19-day cultivation [29]. Interestingly, the productivity of C16 and C18 series did not differ significantly among the salinities investigated [29].

### 5.4. Combined Stress Factors

Compared to one single stress factor, lipid synthesis was further improved by exposing the algal cells to moderately high light in a nitrogen-depleted medium with higher level of Fe^3+^ concentration in *Botryococcus*, indicating the necessarity in investigating the interactive effects of environmental factors on cell growth and biosynthesis of lipids [62]. Culture conditions were traditionally investigated by one-at-a-time strategy in the early studies, meaning varying one factor while keep all others constant. The response surface method has been employed to optimize culture conditions of *Nannochloropsis*, and the results suggested that the maximum growth rate was achieved under 21 °C, 52 μmol/m^2^·s, pH 8.4 and 14.7 VVH of aeration rate [68]. It was reported that a combination of high salinity and high light induced an increase in total fatty acids in *N. oceanica* [59]. Although the beneficial effects of combined stress factors on lipid accumulation have been demonstrated by several reports [59,62,68], the underlying mechanisms are rarely touched.

## 6. Lipid Synthesis Pathway

The biosynthesis and accumulation of storage neutral lipids appear to be a protective reaction in response to stress conditions, yet the underlying lipid metabolism in microalgae remains to be fully understood. The studies on lipid biosynthesis and regulation in *Nannochloropsis* are limited [20,69,70]. Hence, it is of great importance to learn from other well studied algae especially the model alga *Chlamydomonas*.

### 6.1. The Physiological Role of Lipids

Membrane lipids such as phosphatidylcholine (PC), MGDG, DGDG, sulfoquinovosyldiacylglycerol (SQDG), phosphatidylethanolamine (PE) and phosphatidylglycerol (PG) are structural lipids and support the normal cell functions. PUFAs are mainly located in these membrane polar lipids [32,65]. One known function of PUFAs is to scavenge reactive oxygen species (ROS). ROS interacts with the double bonds, causing lipid peroxidation, which may play a role as an intermediate in cell signaling pathways [71,72]. By contrast, the storage neutral lipids (mainly TAGs) accumulated under unfavorable conditions serve as a sink for excessive energy and may be transformed to membrane lipids when rearrangement of photosynthetic apparatus occurs [73].

### 6.2. De Novo Fatty Acid Synthesis

**Figure 4:**
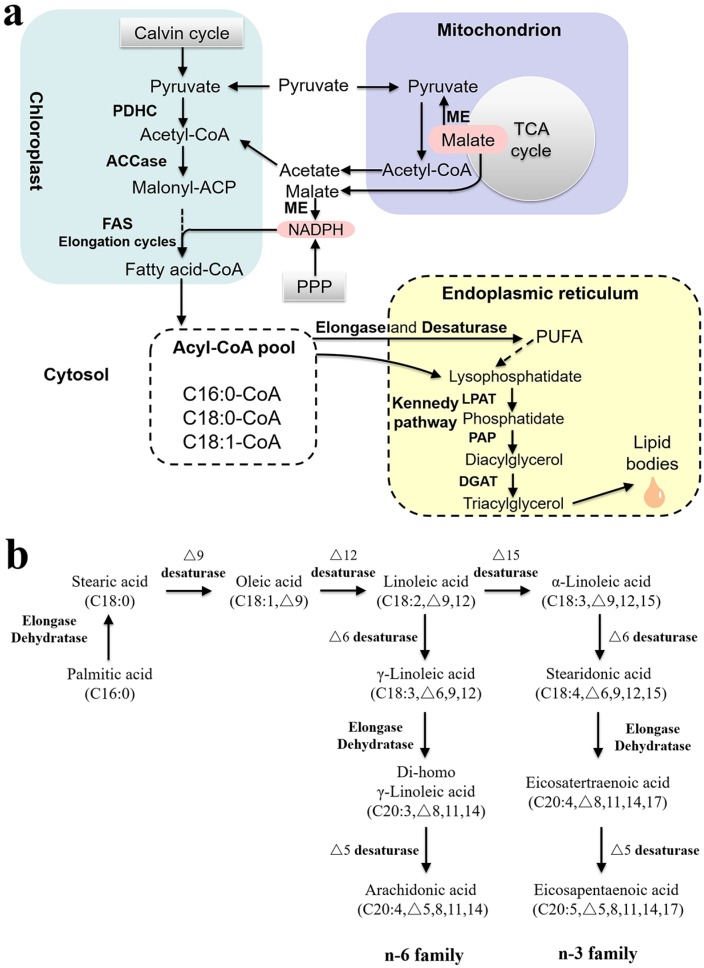
The lipid biosynthesis pathway in *Nannochloropsis*. (**a**) The carbon metabolic pathways related to lipid synthesis; (**b**) The proposed PUFA biosynthesis pathway. PDHC, pyruvate dehydrogenase complex; ACCase, acetyl-CoA carboxylase; FAS, fatty acid synthase; ME, malic enzyme; LPAT, lysophosphatidate acyltransferase; PAP, phosphatidic acid phosphatase; DGAT, diacylglycerol acyltransferase; PUFA, polyunsaturated fatty acid; TCA cycle, tricarboxylic acid cycle; PPP, pentose phosphate pathway.

A solid understanding toward global fatty acid biosynthesis pathway has been established, which is generally divided into three parts (Figure 4a): (1) continuous supply of acetyl-CoA as the precursor and NADPH as the reductant; (2) carboxylation of acetyl-CoA to form malonyl-CoA; (3) acyl chain elongation [74].

The acetyl-CoA produced via pyruvate dehydrogenase complex (PDHC) in chloroplast participates in *de novo* fatty acid synthesis directly, and the plastid seems to be the major site of acetyl-CoA formation. Acetyl-CoA can also be produced from pyruvate via a so-called “PDHC bypass”, in which pyruvate is converted to acetate by pyruvate decarboxylase (PDC) and aldehydedehydrogenase (ALDH) [69]. PDHC in chloroplast bridging the glycolysis and lipid biosynthesis pathways, is considered as a key enzyme to push carbon flux into fatty acid synthesis. Malic enzyme (ME) catalyzes malic acid to generate pyruvate and NADPH. Since 16 mol NADPH is needed to produce 1 mol C18 molecular, the ME catalyzed step is assumed to be essential for the supply of enough reductant [75]. It was reported that the introduction of a gene encoding for ME led to a considerable increase in lipid content in microalgae while having little effect on cell growth [76,77]. Interestingly, low or zero ME activity is detected in *Nannochloropsis* during lipid accumulation [78], which may suggest that NADPH is also supplied by other pathways, such as pentose phosphate pathway (PPP) and cytosolic isocitrate dehydrogenase catalyzed reaction [79].

Once acetyl-CoA is generated, it is catalyzed by acetyl-CoA caboxylase (ACCase) to form malonyl-CoA. This process is generally considered as a rate-limiting step, but Courchesne [74] pointed out ACCase may not be the only rate-limiting enzyme in microalgae. The increased expression of plastidial ACCase correlated positively with the increased fatty acid content over time in *Chromera velia* [80]. An increased substrate pool of malonyl-CoA resulting from the raised ACCase specific activity enhanced TAG accumulation in *N. oculata* [81].

Malonly-CoA enters the elongation of fatty acids catalyzed by the fatty acid synthase (FAS) complex. FAS is a multi-enzyme responsible for the formation of palmitic acid (C16:0) and stearic acid (C18:0) [74].

As shown in Figure 4b, oleic acid (C18:1, *n*-9) is generated from stearic acid (C18:0) by Δ9 desaturase, followed by series of subsequent stepwise desaturation and elongation steps to form *n*-3 PUFA family including EPA [9,12]. EPA linked to diacylglycerol-*N*,*N*,*N*-trimethylhomoserine (DGTS), PE or PC molecules can be imported into the plastid for the incorporation into glycolipids [71,82,83].

A Δ6 desaturase gene and a fatty acid elongase gene were cloned and characterized from *N. oculata* [84,85]. The overexpression of Δ12 desaturase in *N. oceanica* significantly altered fatty acid composition of total lipids and of individual lipid classes, and increased the percentage of linoleic acid (C18:2, *n*-3) and eicosatetraenoic acid (C20:4, *n*-3) [86].

### 6.3. TAG Formation

TAG formation undergoes a series of acylation steps of glycerol-3-phosphate. First, glycerol-3-phosphate is acylated with an acyl-CoA by glycerol-sn-3-phosphate acyl-transferase (GPAT), generating lysophosphatidate. Then lysophosphatidate is acylated with another acyl-CoA to form phophatidate, which is catalyzed by lysophosphatidate acyl-transferase (LPAT). The dephosphorylation of phosphatidate leads to the formation of diacylglycerol (DAG). Finally, DAG accepts a third acyl-CoA to form TAG, catalyzed by diacylglycerol acyltransferase (DGAT) [87,88,89]. This is generally considered as the major pathway for TAG biosynthesis in microalgae [90].

Transcriptomics and lipidomics of *N. oceanica* IMET1 revealed the upregulation of seven putative DGAT genes and downregulation of most genes involved in *de novo* fatty acid synthesis under nitrogen-depleted conditions [69]. Moreover, the pathways of channeling carbon precursors from proteins and carbohydrates into glycerolipid synthesis were enhanced at the transcriptional level under nitrogen-depleted conditions [69]. Phospholipid: diacylglycerol acyltransferase (PDAT) also plays a role in TAG formation by transferring an acyl group from the *sn*-2 position of polar lipids to DAG [91]. PDAT from *Chlamydomonas reinhardtii* (CrPDAT) was predicted to be chloroplast-localized and its presence in the chloroplast was confirmed by proteomic analysis [92]. CrPDAT was upregulated transiently with the degradation of membrane lipids and enhanced TAG accumulation upon stress conditions; moreover, both *in vitro* and *in vivo* experiments demonstrated CrPDAT utilized polar membrane lipids as the substrates for TAG formation [93]. Nevertheless, it is worth noting that CrPDAT contributes to TAG biosynthesis mainly under favorable growth conditions and early stress conditions, which is different from the roles of DGAT [93]. Bioinformatics analysis revealed the presence of a PDAT gene copy in the *Nannochloropsis* genome [70], yet its function and *in vivo* roles remain to be explored.

The DAG moiety for TAG assembly can be either directly deprived from the *de novo* synthesis or converted from membrane lipids. It was reported that only about 7% TAG utilized the *de novo* synthesized DAG directly for assembly while the remainder utilized the PC-derived DAG [94], indicating a role of PC in TAG biosynthesis in higher plants. Similarly, PC is present in *Nannochloropsis* and it may serve as the provider of a DAG moiety for TAG. TAG accumulates and is packed into LDs. The investigation of LD formation and turnover in *Nannochloropsis* disclosed an abundant hydrophobic lipid droplet surface protein with unique primary sequence [95]. This protein abundance parallelled TAG content, indicative of its regulation on TAG-rich LD formation.

## 7. Genetic Engineering Approaches for Lipid Production in *Nannochloropsis*

The lack of ideal strains which can be selectively optimized for both high biomass and high lipid content hampers the large scale cultivation of *Nannochloropsis*. Genetic engineering, represents an efficient approach towards offering huge potential in modifying microalgae for lipid production. For common laboratory model algae, such as *Chlamydomonas reinhardtii* and *Phaeodactylum tricornutum*, the transformation protocols have been available for years [77,96]. However, the industrial application of these strains are limited due to the relatively low lipid productivity. Successful genetic transformation of *Nannochloropsis* have been reported in several papers (Table 5). Electroporation is used as the main transformation method, and both endogenous and foreign promoters are successfully applied to drive the overexpression of target genes [20,97,98,99,100]. The examination of successful *N. gaditana* transformation was also done after 4–5 months of growth with antibiotic selection [19]. The further investigation on stable transgene integration and expression in *Nannochloropsis* is still needed based on earlier attempts as well as the known genome sequences.

## 8. Future Prospects of *Nannochloropsis*

Even though microalgae have long been considered as the promising feedstocks for biodiesel, it cannot be ignored that the economics of microalgal biodiesel production need to be significantly improved. At present, most studies focus on investigating the effects of environmental factors and nutrients on lipid accumulation in *Nannochloropsis*, but the efforts on the understanding of lipid biosynthesis regulation and the use of efficient genetic tools to improve cellular TAG or PUFA biosynthesis were less touched. With the growing importance of *Nannochloropsis* in the production of biodiesel and other value-added products, it is necessary to identify the molecular mechanisms and metabolic pathways underlying the lipid synthesis in order to facilitate the application of new genetic techniques to the algae.

Quantitative real-time reverse transcription PCR (RT-PCR) has been utilized to identify and test the potential genes in *Nannochloropsis* sp. [101]. The results showed that the best reference genes differed depending on the environmental conditions, and a combination of actin and beta-tubulin would be appropriate as a reference panel for normalizing gene expression data across all the treatments [101]. The changes in transcript abundance in *Nannochloropsis* sp. following nitrogen deprivation provided a potential source for molecular mechanisms exploration of lipid accumulation. Besides, a set of simple sequence repeat motifs was identified from the expressed sequence tags (EST), which would provide useful genetic markers for further genetic analysis [102]. In addition, the supplying carbon precursors and energy related genes, such as those encoding components of PDHC, glycolysis, PDHC bypass and sets of specific transporters, were substantially upregulated under nitrogen-depleted conditions, resulting in increased overall TAG production in *N. oceanica* [69]. Moreover, genes involved in the citric acid cycle and β-oxidation in mitochondria were greatly enhanced to utilize the carbon skeletons derived from membrane lipids and proteins to produce additional TAG or its precursors [69]. These studies will provide useful information allowing for the development of more sophisticated genetic engineering for future applications.

Although the changes in gene expression level have been reported in several *Nannochloropsis* species, the metabolic mechanism is still poorly understood. Those key points and possible rate-limiting steps in the pathway (Figure 4) are consequently the putative targets for genetic engineering, as evidenced by studies on other organisms [12]. Given that most of the genes involved in *de novo* fatty acid synthesis were downregulated (including ACCase and FAS) while the total glycerolipid content increased five fold following nitrogen starvation, it was speculated that these enzymes may be present in excess for fatty acid synthesis [69]. In this case, limited success may be obtained by overexpressing these genes involved in *de novo* fatty acid synthesis. Photosynthetic carbon precursors, multiple routes for acetyl-CoA synthesis and Kennedy pathway might be promising candidates for genetic engineering. On the other hand, the lipogenic capacity of *Nannochloropsis* strains under investigation seems to be restricted by the limited NADPH synthesis due to the low ME activity, thus the key enzymes supplying NADPH are also potential targets of manipulation. In addition, the pathways for the conversion of membrane lipids to neutral lipids have been rarely reported and are worthy of deep investigation. For EPA production by *Nannochloropsis*, efforts should be paid to those elongases and desaturases responsible for the biosynthesis of long-chain ω-3 PUFAs [9].

## 9. Conclusions

The role of *Nannochloropsis* as a potential microalgal cell factory for lipid production has received ever-increasing attention in recent years. The integrated production of biofuels and the high-value product EPA, coupled with the environmentally beneficial applications like flue gas and wastewater treatment represents a promising direction toward cost-effective production process of *Nannochloropsis*, which requires close collaboration between biologists and engineers. Research and development priority should be given to the better understanding of molecular mechanism for lipid metabolism and the development of a more stable and robust genetic toolkit for *Nannochloropsis* to enable this organism to be a true cell factory for the production of lipids. To this end, comprehensive analyses of certain potential strains are underway via genomic, transcriptomic, proteomic, lipidomic, and metabolomic approaches. The availability of those omics data will uncover the biological implications and pave the future tailored manipulation of *Nannochloropsis* for broader industrial applications.

## Figures and Tables

**Table 1:** Lipid content of selected microalgal strains.

Species	Total Lipid Content (% of DW)	Neutral Lipid Content (% of Total Lipid)	Ref.
*Nannochloropsis*	37–60	23–58	[16]
*Isochrysis*	25–33	80	[1,22]
*Dunaliella salina*	23	30	[23,24]
*Haematococcus pluvialis*	16–35	50–59	[25]
*Neochloris oleoabundans*	2–47	23–73	[26]
*Phaeodactylum tricornutum*	20–30	-	[27]
*Crypthecodinium cohnii*	20	-	[1]
*Spirulina platensis*	7.6–8.2	-	[28]
*Tetraselmis maculata*	8	-	[27]
*Scenedesmus obliquus*	12–14	-	[27]

**Table 2:** The fatty acid profile (% total fatty acid) of selected *Nannochloropsis* strains.

*Nannochloropsis* Species	C14:0	C16:0	C16:1	C16:2	C18:0	C18:1	C18:2	C18:3	C20:1	C20:4	C20:5	Ref.
*N. oculata* CS179		26.7	26.6		0.6	5.9	5.3	0.1		7.1	20.2	[29]
*N. oceanica* CCMP531	4.5	45.9	22.7		0.6	22.2	0.7	0.5		2.5	2.9	[16]
*N. oculata* CCMP529	2.1	29.1	28.3		1.9	22.8	2.6	1.6		6.2	5.4	[16]
*N. limnetica* CCMP505		16.6	2.9	3.1	4.6	31.5	23.8	17.5				[16]
*N. granulata* CCMP525	2.4	26.2	24.0		3.3	28.5	4.7	1.6		4.5	4.8	[16]
*N. gaditana* CCMP527	2.7	39.2	24.1		3.1	14.2	4.7	1.6		4.5	4.7	[16]
*N. salina* CCMP537	3.3	32.2	25.4	3.0	2.5	15.5	3.0	0.6		3.6	10.9	[16]
*N. salina* CCMP1176	2.1	32.0	30.0		3.2	9.4	2.6	0.9		7.2	12.7	[16]
*Nannochloropsis* sp.	4.3	24.6	30.2		1.1	11.0	1.9		1.5	21.8		[30]
*Nannochloropsis* sp.	4.0	21.8	25.8			4.2	2.5			6.5	33.7	[31]

**Table 3:** Examples of *Nannochloropsis* characterized by EPA production.

Species	EPA Content (% DW)	Ref.
*N. gaditana*	4.3	[37]
*N. oceanica* CY2	4.4–5.1	[38]
*N. oceanica* CY2	5.5	[35]
*N. salina*	1.1–3.5	[15]
*N. oceanica* IMET1	2.7–5.2	[39]
*N. oculata*	2–3	[40]
*Nannochloropsis* sp.	12	[36]
*Nannochloropsis* sp.	5–6	[41]
*Nannochloropsis* sp.	3–6	[30]
*Nannochloropsis* sp.	4	[31]
*Nannochloropsis* sp.	2–4	[42]

**Table 4:** Reported *Nannochloropsis* cultivation for waste water or flue gas treatment.

*Nannochloropsis* Species	Waste Water Sources	Flue Gas Sources	Specific Growth Rate (/day)	Lipid Content (% DW)	Ref.
*N. salina*	Anaerobic digestion effluent		0.3–0.6	21–36	[43]
*N. salina*	Wastewater Reclamation Facility/ Stream from dewatering anaerobically digested sludge				[44]
*N. salina*	Anaerobic digestion effluent		0.04–0.15	26–32	[45]
*N. oceanica*		Coal-fired power plants	0.05–0.07	21–28	[46]
*Nannochloropsis* sp.	Municipal wastewater	Filtered compressed gas (15% CO_2_)	0.52	33	[47]
*N. limnetica*		Rice husk emission			[48]

**Table 5:** Reported *Nannochloropsis* species for genetic modification.

*Nannochloropsis* Species	Reporter or Marker Gene	Promoter	Selection	Transformation Method	Ref.
*N. gaditana*	Bleomycin resistance	TUB, UEP, HSP	Bleomycin	Electroporation	[19]
*N. salina*	GUS	TUB	Zeocin	Electroporation	[99]
*N. oculata*	Fish growth hormone	HSP, RUBISCO SSU 2	Ampicillin	Electroporation	[97]
*N. oculata*	Bovine lactoferricin	HSP, RUBISCO		Electroporation	[100]
*N. oceanica*	Δ12 desaturase	LDSP	Uracil auxotrophy	Electroporation	[86]

TUB, β-tubulin; UEP, ubiquitin extension protein; HSP, heat shock protein; GUS, β-glucoronidase; LDSP, lipid droplet surface protein.
